# Supplementary material for: Proteomics and constraint-based modelling reveal enzyme kinetic properties of Chlamydomonas reinhardtii on a genome scale
Source: Nat Commun. 2023 Aug 8;14:4781. doi: 10.1038/s41467-023-40498-1 (PMC10409818; doi:10.1038/s41467-023-40498-1)
Supplement: Supplementary file 1 — Supplementary Information [file 41467_2023_40498_MOESM1_ESM.pdf]

Supplementary Information accompanying the Research Article

Proteomics and constraint-based modelling reveal enzyme kinetic properties of *Chlamydomonas reinhardtii* on a genome scale

Marius Arend[1,2,3], David Zimmer[4], Rudan Xu[1,2], Frederik Sommer[5], Timo Mühlhaus[4], Zoran Nikoloski[1,2,3,*]

[1]Bioinformatics, Institute of Biochemistry and Biology, University of Potsdam, Potsdam, Germany
[2]Systems Biology and Mathematical Modelling, Max Planck Institute of Molecular Plant Physiology, Potsdam, Germany
[3]Bioinformatics and Mathematical Modeling Department, Center of Plant Systems Biology and Biotechnology, 4000 Plovdiv, Bulgaria
[4]Computational Systems Biology, TU Kaiserslautern, 67663 Kaiserslautern, Germany
[5]Molecular Biotechnology & Systems Biology, TU Kaiserslautern, Kaiserslautern, Germany
*Corresponding author. Email: nikoloski@mpimp-golm.mpg.de

This PDF file includes:

Figs. S1 to S6
Tables S1 & S2

The following materials are included as separate .xlsx files:

Supplementary Data 1 to 4

Supplementary Figure

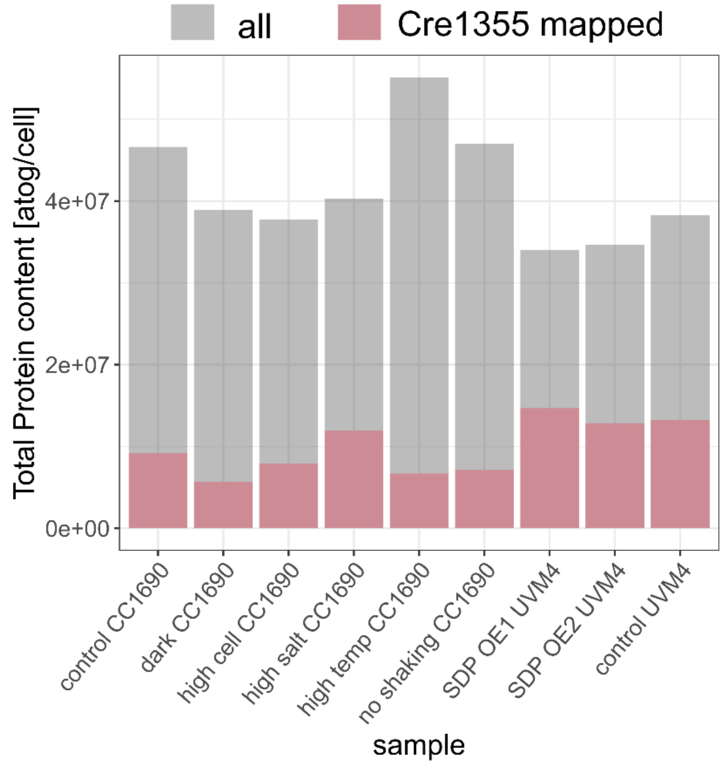

Figure S1. Total protein mass of proteomics data. Bars indicate the median sample-specific protein mass as calculated by the GECKO measureAbundance() function. The dark red bar illustrates the subset corresponding to enzymes present in the Cre1355 model. Plotted values are provided in the source data.

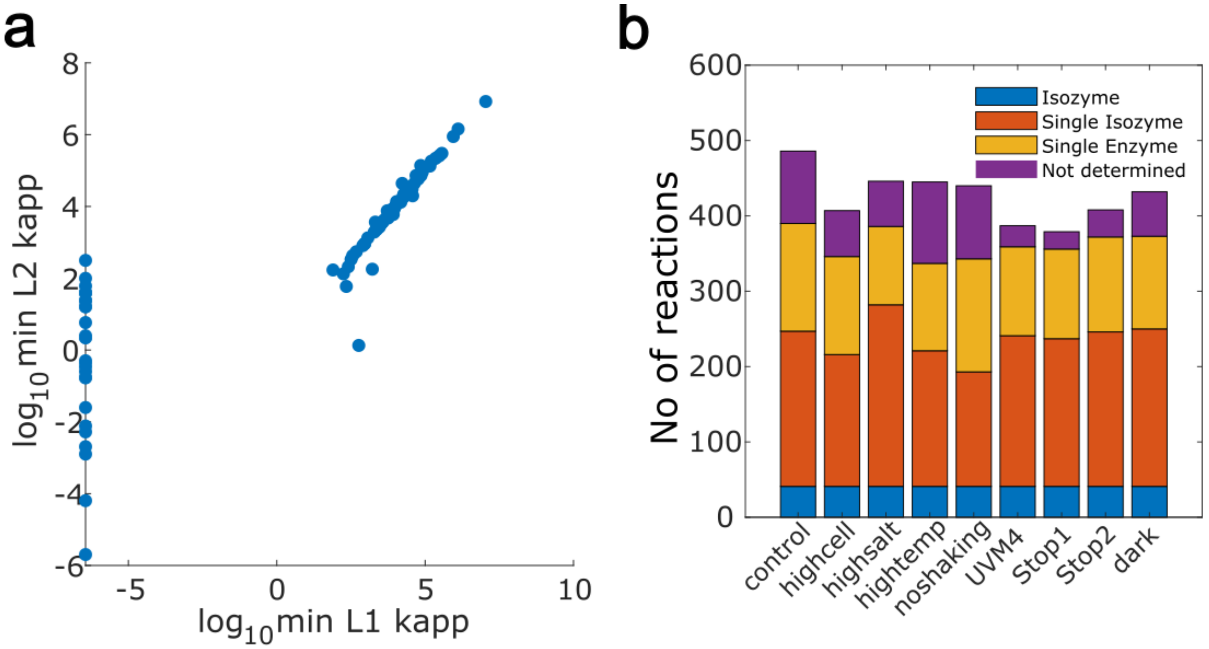

Figure S2. Properties of k_{app} calculated for isoenzyme reactions. (a) Scatterplot of enzyme-specific k_{app} calculated for reactions with multiple expressed isoenzymes. The x-axis provides the estimates obtained by the linear formulation (i.e. minimizing the ℓ^1- norm of the error term δ). The minimal values correspond to the lower bound of $\epsilon = 3.6 \cdot 10^{-7}\ h^{-1}$. The y-axis provides the values estimated by the quadratic formulation. **(b)** Stacked barplot indicating the number of reactions for which single enzymes or (multiple) isoenzymes have been quantified. The category "not determined" originates from reactions with available protein abundance that do not carry flux.

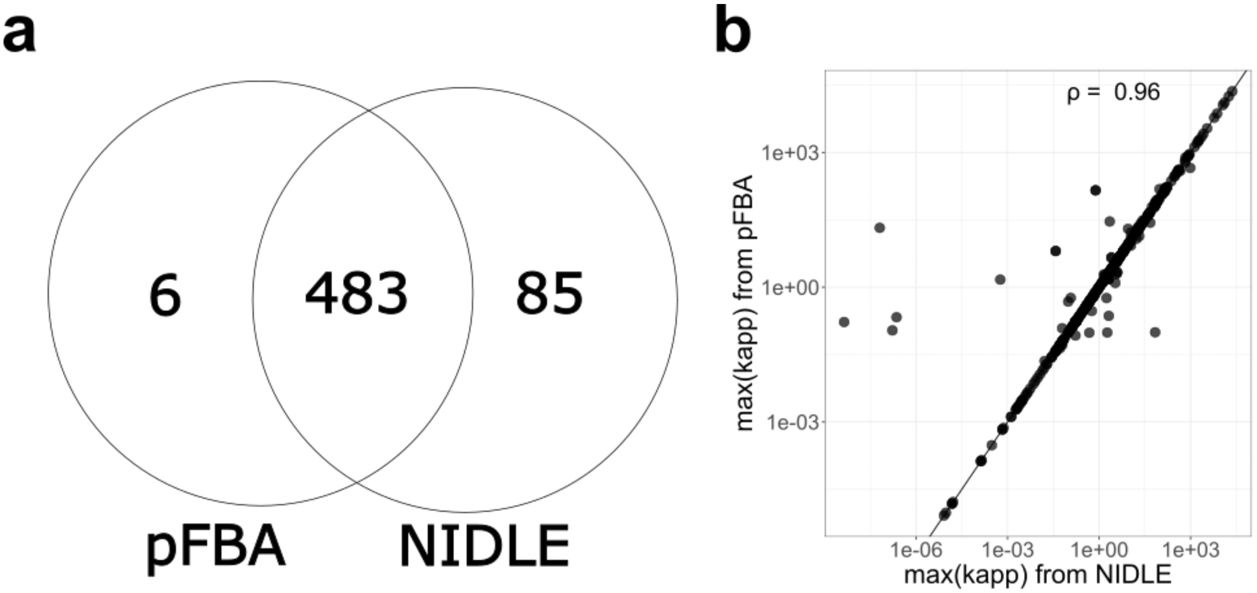

Figure S3. Comparison between pFBA and NIDLE based k_{app}^{max} estimates: (a) Venn Diagram of enzyme catalyzed reactions for which k_{app}^{max} could be estimated by the two approaches. **(b)** Scatterplot of k_{app}^{max} values for the intersect in reactions. Values are plotted in log-scale. ρ: Spearman correlation of log-transformed values. Plotted values are provided in the source data.

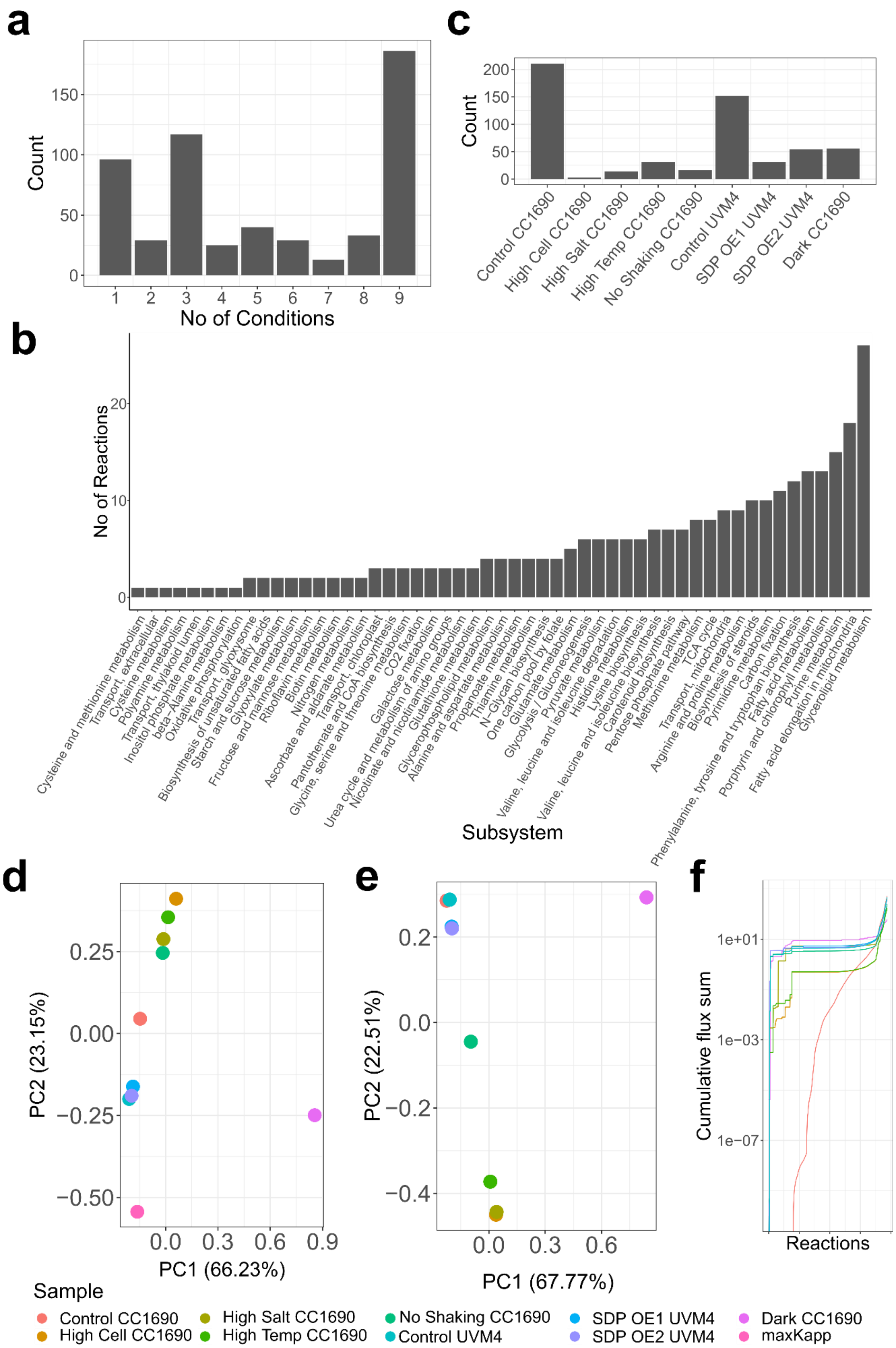

a
Count
No of Conditions
c
Count
Control CC1690
High Cell CC1690
High Salt CC1690
High Temp CC1690
No Shaking CC1690
Control UVM4
SDP OE1 UVM4
SDP OE2 UVM4
Dark CC1690
b
No of Reactions
Subsystem
Cysteine and methionine metabolism
Transport, extracellular
Cysteine metabolism
Polyamine metabolism
Transport, thylakoid lumen
Inositol phosphate metabolism
beta-Alanine metabolism
Oxidative phosphorylation
Transport, glyoxysome
Biosynthesis of unsaturated fatty acids
Starch and sucrose metabolism
Glyoxylate metabolism
Fructose and mannose metabolism
Riboflavin metabolism
Biotin metabolism
Nitrogen metabolism
Ascorbate and aldarate metabolism
Transport, chloroplast
Pantothenate and CoA biosynthesis
Glycine, serine and threonine metabolism
CO2 fixation
Galactose metabolism
Urea cycle and metabolism of amino groups
Nicotinate and nicotinamide metabolism
Glutathione metabolism
Glycerophospholipid metabolism
Alanine and aspartate metabolism
Propanoate metabolism
Thiamine metabolism
N-Glycan biosynthesis
One carbon pool by folate
Glutamate metabolism
Glycolysis / Gluconeogenesis
Pyruvate metabolism
Valine, leucine and isoleucine degradation
Histidine metabolism
Lysine biosynthesis
Valine, leucine and isoleucine biosynthesis
Carotenoid biosynthesis
Pentose phosphate pathway
Methionine metabolism
TCA cycle
Transport, mitochondria
Arginine and proline metabolism
Biosynthesis of steroids
Pyrimidine metabolism
Carbon fixation
Phenylalanine, tyrosine and tryptophan biosynthesis
Fatty acid metabolism
Porphyrin and chlorophyll metabolism
Purine metabolism
Fatty acid elongation in mitochondria
Glycerolipid metabolism
d
PC2 (23.15%)
PC1 (66.23%)
e
PC2 (22.51%)
PC1 (67.77%)
f
Cumulative flux sum
Reactions
Sample
Control CC1690
High Cell CC1690
High Salt CC1690
High Temp CC1690
No Shaking CC1690
Control UVM4
SDP OE1 UVM4
SDP OE2 UVM4
Dark CC1690
maxKapp

Figure S4. Distribution of k_{app} values. (**a**) Histogram of the number of conditions in which k_{app} was calculated for the homomeric or isoenzyme-catalyzed reactions in the iCre1355 model. (**b**) Number of homomeric or isoenzyme-catalyzed reactions for which the maximum observed k_{app} was found in the respective condition. (**c**) Barplot giving the distribution of reactions for which a k_{app} value could be obtained in all of the 9 condition (rightmost bar in (**a**)) over the different metabolic subsystems indicated at the x axis. (**d**) Principal component analysis of log-transformed k_{app} values per condition and the vector of maximum k_{app} values. (**e**) Principal component analysis of log-transformed flux values of the NIDLE solution per condition. Only reactions that were assigned non-zero values in all conditions were considered in the PCAs. (**f**) Cumulative sum of flux vector v in the NIDLE solution, ordered by ranks in the Control CC1690 flux solution. Plotted values of panels **a, b, c, f** are provided in the source data.

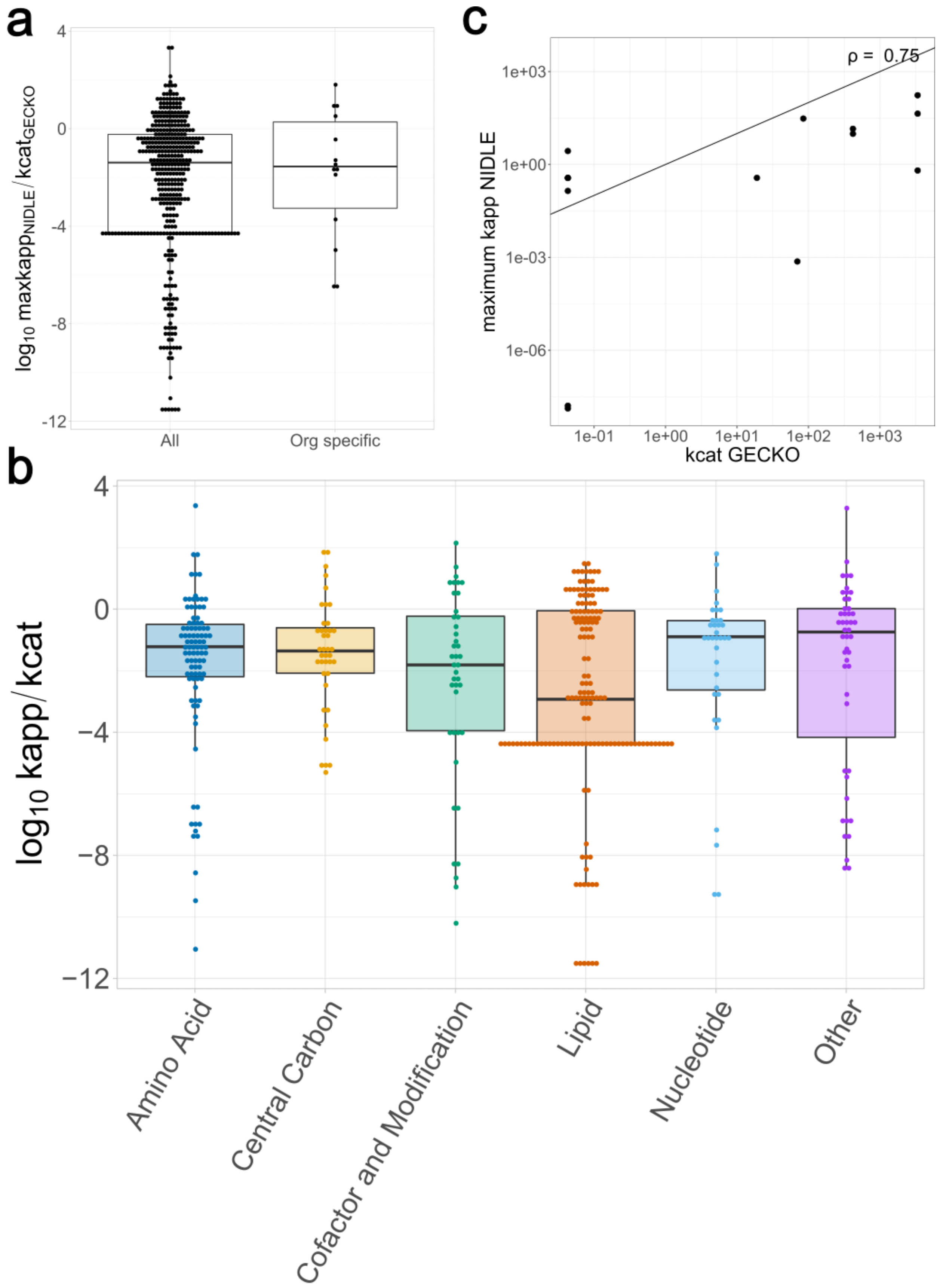
a
log10 maxkappNIDLE/kcatGECKO
4
0
-4
-8
-12
All
Org specific
c
ρ = 0.75
maximum kapp NIDLE
1e+03
1e+00
1e-03
1e-06
1e-01
1e+00
1e+01
1e+02
1e+03
kcat GECKO
b
log10 kapp/kcat
4
0
−4
−8
−12
Amino Acid
Central Carbon
Cofactor and Modification
Lipid
Nucleotide
Other

67

Figure S5. Distributions and correlation of organism-specific GECKO k_{cat} values and NIDLE k_{app}^{max} values. **(a)** Boxplot of the $\log_{10}$-fold difference between NIDLE k_{app}^{max} and GECKO k_{cat}. For all enzymatic reactions ("All") or only for reactions with GECKO k_{cat} values of *C. reinhardtii* enzymes ("Org specific"). The boxes mark the interquartile range with the bold bar giving the median. Lines give the last data point within 1.5 times of the interquartile range. Points in the overlay give the single observations and have been binned. (**b**) Boxplot of the $\log_{10}$-fold difference between NIDLE k_{app}^{max} and GECKO k_{cat} values, subdivided by metabolic macrosystem. The boxes mark the interquartile range with the bold bar giving the median. Lines give the last data point within 1.5 times of the interquartile range. Points in the overlay give the single observations and have been binned. Group sizes: Amino Acid: 93, Central Carbon: 40, Cofactor and Modification: 41, Lipid: 149, Nucleotide: 35, Other: 47 (**c**) Scatter plot of enzymatic reactions that got assigned organism specific literature k_{cat} values by GECKO. ρ: Spearman correlation. Raw values are provided in the source data.

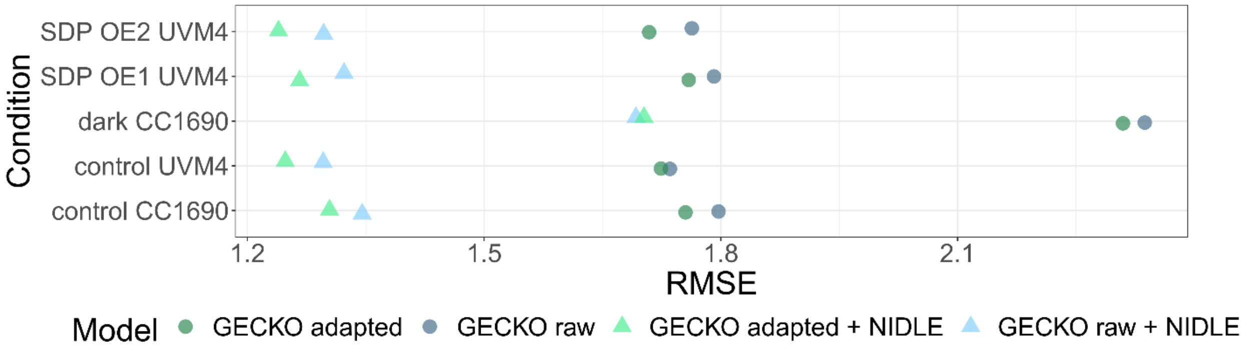

Figure S6. RMSE of enzyme usage predictions in unseen conditions. RMSE of predicted enzyme usage based on pcGEMs and observed enzyme abundance in QConCat data set in log10 scale. The tested condition was not considered when calculating the k_{app}^{max} values from NIDLE. Plotted values are provided in the source data.

Supplementary Tables

Table S1: Parameters used in GECKO enzyme pool constrain. Table contains the GECKO protein pool parameters of models created in this study. f: Proteome enzyme partition; σ: average enzyme saturation coefficient; Ptot: Total protein content (29).Only one model per two replicates in the data set of Imam et al. (18) was created since each two replicates had the same growth rate. [a]The f factor is different for heterotrophic models since GECKO toolbox removed blocked enzyme catalyzed reactions in this condition. Therefore, the number of enzymes in the model differ.

	Autotrophic Rep1	Autotrophic Rep3	Mixotrophic Rep1	Mixotrophic Rep3	Heterotrophic Rep1
**f[a]**	**0.42**	**0.42**	**0.42**	**0.42**	**0.39**
**σ**	**0.45**	**0.52**	**0.52**	**0.5**	**0.23**
**Ptot [g/gDW]**	**0.261**	**0.261**	**0.303**	**0.303**	**0.222**

Table S2 : Flux bounds differing from model bounds provided by Imam et al.[27]. Acetate uptake rate was estimated from growth rate in mixotrophic conditions and taken from chemostat measurements of Imam et al.[27] in heterotrophic conditions. Light intensity was identical to mixotrophic chemostat so bounds were not adapted.

**Condition**	**Growth rate, μ [h^-1]**	**Acetate Uptake EX_ac_e [mmol/gDWh]**	**iCre1355 Model**
Control CC1690	0.11	3.57	mixo
highcell CC1690	0.02	0.99	mixo
highsalt CC1690	0.02	0.99	mixo
hightemp CC1690	0.02	0.99	mixo
noshaking CC1690	0.04	1.20	mixo
Control UVM4	0.0937	2.72	mixo
Stop1 UVM4	0.0864	2.35	mixo
Stop2 UVM4	0.0837	2.22	mixo
dark CC1690	0.02	1.60	hetero
