# Supplementary material for: Proteomics and constraint-based modelling reveal enzyme kinetic properties of Chlamydomonas reinhardtii on a genome scale
Source: Nat Commun. 2023 Aug 8;14:4781. doi: 10.1038/s41467-023-40498-1 (PMC10409818; doi:10.1038/s41467-023-40498-1)
Supplement: Supplementary file 3 — Description of Additional Supplementary Files [file 41467_2023_40498_MOESM3_ESM.pdf]

Description of Additional Supplementary Files

Supplementary Data 1. Median of protein abundancies from triplicates [amol/cell].

Supplementary Data 2. Reaction wise kapp values calculated from NIDLE [s^-1]. Reaction Ids from iCre1355 (19) have been added a suffic "_f", "_b" if a reversible reaction was split forward and backward reaction.

Supplementary Data 3. Experimental parameters and physiological measurements of CC1690 cultures analysed by protein mass spectrometry.

Supplementary Data 4. Enzyme wise kapp values [s^-1]. For promiscous enzymes the maximum of all reaction-wise kapps was calculated in each condition.
